# Discovery of novel immunotherapeutic drug candidates for sciatic nerve injury using bioinformatic analysis and experimental verification

**DOI:** 10.3389/fphar.2022.1035143

**Published:** 2022-11-07

**Authors:** Shengyou Li, Beibei Yu, Xue Gao, Yi Zheng, Teng Ma, Yiming Hao, Haining Wu, Bin Wei, Yitao Wei, Zhuojing Luo, Bing Xia, Jinghui Huang

**Affiliations:** ^1^ Department of Orthopedics, Xijing Hospital, Fourth Military Medical University, Xi’an, China; ^2^ Department of Neurosurgery, The Second Affiliated Hospital of Xi’an Jiao Tong University, Xi’an, China

**Keywords:** sciatic nerve injury, immune infiltration, trichostatin a, basement membranes genes, bioinformatics

## Abstract

Inflammation following nerve injury and surgery often causes peripheral nerve adhesion (PNA) to the surrounding tissue. Numerous investigations independently examined the prevention or inhibition of PNA, however, an intervention targeting macrophages has not been fully elucidated. Basement membrane (BM) genes are known to modulate central nervous system (CNS) inflammation, however, their activities in the peripheral nervous system (PNS) remains undiscovered. In this report, we carried out weighted correlation network analysis (WCNA) to screen for principal sciatic nerve injury (SNI) module genes. Once an association between the module and BM genes was established, the protein–protein interaction (PPI) and immune infiltration analyses were employed to screen for relevant BM-related immune genes (Itgam, SDC1, Egflam, and CD44) in SNI. Subsequently, using the Drug SIGnatures (DSigDB) database and molecular docking, we demonstrated that Trichostatin A (TSA) interacted with key immune genes. TSA is known to enhance M2 macrophage expression and attenuate fibrosis. Nevertheless, the significance of the epigenetic modulation of macrophage phenotypes in dorsal root ganglion (DRG) is undetermined after SNI. In this article, we examined the TSA role in fibrogenesis and macrophage plasticity associated with DRG. We revealed that TSA enhanced M2 macrophage aggregation, inhibited fibroblast activation, and improved sciatic nerve regeneration (SNR) and sensory functional recovery (FR) after SNI. In addition, TSA suppressed M1 macrophages and enhanced M2 macrophage invasion within the DRG tissue. Furthermore, TSA dramatically reduced IL-1β and TNFα levels, while upregulating IL-10 level. In summary, this research revealed for the first time that TSA alleviates fibrosis in DRG by promoting an M1 to M2 macrophage transition, which, in turn, accelerates SNR.

## Introduction

Peripheral nerve injury (PNI) is a widespread disease brought on by accidental trauma, infection, neoplasm, surgery or inflammatory disorders. This is a global phenomenon that affects millions of individuals worldwide. As such, it introduces a heavy social burden with prolonged disability and medical expenditure (Yu et al., 2015). Moreover, peripheral nerve damage can impair motor and sensory function and can permanently cause tissue atrophy (Chen et al., 2019). Even though the mammalian adult peripheral nervous system can potentially experience spontaneous recovery, often the recovery is complicated and persistent, thus producing undesirable outcomes (Chen et al., 2015). Nerve scarring is one of the main causes that limit peripheral nerve regeneration (PNR) (Atkins et al., 2006). At present, there is no optimal intervention that corrects peripheral nerve adhesions (PNAs). Moreover, there is no routine anti-scarring medication employed in clinical practice. Till now, the most frequent treatment plans involve surgical adhesiolysis, scarred tissue resection around the nerve (external neurolysis), and the epifascicular region removal from the epineurium (internal neurolysis). Unfortunately, the benefits are often temporary, as adhesions often re-occur (Lemke et al., 2018). Surgical adhesiolysis is a temporary stopgap--not a real solution that inhibits nerve fibrosis formation. To treat or suppress PNA, it is critical to explore PNA pathogenesis-associated pathways.

It is well known that fibrosis develops due to abnormal extracellular matrix (ECM) growth. The ECM is either arranged as basement membranes (BMs) which nourish the surrounding cellular layers, or as loose interstitial matrix. It not only provides a scaffold, but also modulates organ shape, and recruits growth factors and enzymes for regulated out-in signal transmission. Matrix formation and degradation is strictly modulated in order to maintain organ function. Moreover, excess matrix aggregation is related to a myriad of human diseases (Randles et al., 2021). In the western world, 45% of patient mortality is correlated with organ fibrosis. Therefore, it is critical to identify modulators of excess matrix to enhance strategies that accelerate PNR.

In recent years, multiple reports suggested that inflammation strongly regulates nerve adhesion (Yao et al., 2018; Bu et al., 2020). Being chief inflammatory cells, macrophages modulate tissue injuries in all categories of repair and fibrosis, including PNA (Wynn and Ramalingam, 2012; Lan et al., 2017). Generally, activated macrophages can be found in two distinct forms: proinflammatory (M1) and anti-inflammatory (M2) (Oliveira et al., 2013). M1 synthesizes inflammation promoters after tissue injury (Cao and He, 2013). Alternately, there is no consensus on the M2 function. It is speculated that M2 has multiple subforms with differential functions (Kadl et al., 2010). The regulatory macrophage (Mreg) is widely known as the tissue repairing macrophage, which produces modulatory factors that suppress inflammation and sustain homeostasis (Gordon, 2003). Mreg deficiency therefore generates a chronic injury phenotype, which eventually results in PNA (Murray, 2017; Chandrasekaran et al., 2019). Hence, suppressing the inflammatory response while accelerating Mreg polarization can potentially abrogate PNA.

Herein, we employed weighted correlation network analysis (WGCNA) to analyze key SNI genes. Following intersection with BMs genes, we identified key immune-related CGs (common genes) using protein–protein interaction and immune infiltration analyses. Using the DSigDB database, we next predicted that Trichostatin A (TSA) was a potential target of these genes. Following the experimental validation of an SNI model, we subsequently revealed that TSA inhibited neural inflammation and fibrosis, which, in turn, improved PNR, as well as motor and sensory functional recoveries. Together, this study revealed that the TSA-based anti-inflammatory effects were crucial for the inhibition of neural scarring and improvement of PNR.

## Materials and methods

### Data sampling

The National Center for Biotechnology Information (NCBI) Gene Expression Omnibus (GEO) database (https://www.ncbi.nlm.nih.gov/geo/) was employed for the SNI-based gene profile information. The raw data was acquired from the murine sciatic nerve tissue gene chips GSE96051 and GSE161342. “A basement membrane discovery pipeline uncovers network complexity, regulators, and human disease associations” (PMID: 35584218) was utilized to identify 224 BM genes (Jayadev et al., 2022), which are summarized in Supplementary Table S1. GSE96051 is composed of 4 normal murine sciatic nerve and 5 murine SNI transcript information (Larhammar et al., 2017); and GSE161342 included 12 murine sciatic nerve tissue samples, including 3 IPA samples, 3 IPA-SNC samples, 3 PBS samples and 3 PBS-SNC samples. We used PBS group and PBS-SNC group for the subsequent analysis. All data were extracted, processed, then normalized.

### Data processing and differential expression analysis

All data analyses and visualization were carried out in R version 4.1.0 (R Foundation for Statistical Computing, Vienna, Austria). Differences in the chip profile standardization were assessed *via* the “limma” package (Ritchie et al., 2015). Batch effects were eliminated *via* the “SVA” package (Leek et al., 2012). Correction testing was carried out *via* the Bayes method multiple, and differentially expressed genes (DEGs) were identified according to | log2FC | >0.58 and *p* < 0.05. Lastly, DEG cluster analysis was performed and a heatmap and volcano plot were generated *via* the “pheatmap” and “EnhancedVolcano” packages.

### Weighted correlation network analysis

The “WGCNA” package was used to assess interactions between SNI genes. The cutreeDynamic function pruned the gene hierarchical clustering dendrograms into co-expression modules, and the associated modules were subsequently merged. The module eigengenes (ME) dissimilarity was computed *via* moduleEigengenes function, and the association between individual modules and sample traits were further examined. Modules associated with the strongest positive and negative correlations were chosen for detailed analyses.

### Screening of the basement membrane-linked genes

Overall, 224 BMs-linked genes were acquired from the supplementary publication by Jayadev et al. (2022). The “homologene” package was used to convert the homologene from human to mouse. To screen BMs-linked genes, Venn analysis was employed to establish the intersection of the two gene sets, namely, the WGCNA-based chief module genes and BMs-linked genes. Gene Ontology (GO), Kyoto Encyclopedia of Genes and Genomes (KEGG) network analyses were carried out *via* the “clusterProfiler” package to screen for SNI associated signaling networks and physiological function.

### Generation of a protein-protein interaction axis and hub gene screening

The STRING Database (https://string-db.org/) is generally utilized to predict gene significance, analyze gene lists, and prioritize genes for functional assays. Herein, PPI was generated with the STRING database. The medium confidence (0.4) was the minimum required interaction score. Next, we employed the Cytoscape software to assess the module data, and the strictly related modules in PPI to estimate the association between DEG-encoded proteins. Lastly, we identified core BM-related genes in SNI using the MCODE plugin in the Cytoscape software.

### Immune state analysis

This investigation employed CIBERSORT (R package) to assess the immunologic profile of SNI tissues. Values with a *p*-value < 0.05 were selected and the percentage of 22 immune cells were computed. Lastly, the Spearman’s correlation analysis was employed to extensively examine the identified genes.

### Peripheral nerve injury modeling

Adult male Sprague–Dawley (SD) rats (200–250 g) were provided by the Laboratory Animal Center of the Fourth Military Medical University, and were maintained according to the ethical guidelines set by the NIH Guide. In addition, we received ethical approval for this work from the aforementioned university. In brief, five rats were maintained in individual cages in a temperature-regulated facility (23 ± 2°C; 35%–60% humidity; 12 h light/dark cycle).

The rat SNI model was established as reported in earlier publications, with minor modifications (Li et al., 2017). We employed vascular clips (30 g for 50 s; Oscar, Guangzhou, China) to compress the sciatic nerve at 7 mm proximal to the sciatic nerve trifurcation. Subsequently, we closed the wound with nondegradable sutures. Next, animals were randomly placed in two distinct groups: 1) SNI; and 2) SNI + TSA (*n* = 12 per group). Sham rats were provided with the same surgery. However, they did not undergo SNI. The TSA rats were intraperitoneally administered TSA (2 mg/kg of body weight) for 7 days (once/day) after injury. All rats received the same volume of saline solution.

### Biochemical analysis

DRG tissues were homogenized in PBS (10 mg tissue to 100 μl PBS), then centrifuged at 12,000 for 20 min at 4°C. Protein quantification was done *via* the Bradford test (Bio-Rad, Hercules, CA, United States). Rat ELISA Kits from Mybiosource (San Diego, CA, United States) and Cayman (Ann Arbor, Michigan, United States) were employed for the inducible nitric oxide synthase (iNOS, Cat. #MBS023874) and prostaglandin E2 (PGE2, Cat. #514,010) measurements, respectively. NADPH oxidase 1 (NOX1, Cat. #CSBEL015959RA), cyclooxygenase-2 (COX-2, Cat. #CSB-E13399r), 5-lipoxygenase (5-LOX, Cat. #CSB-E16982r), catalase (Cat. #CSBE13439r), TNF-α (Cat. #CSB-E11987r), and NF-κB (Cat. #CSBE13148r) were examined *via* Cusabio (Houston, TX, United States) ELISA Kits, based on kit directions.

### Western blot analysis

DRG tissue lysis was done using RIPA buffer (Beyotime) with phosphatase and protease inhibitors for over 5 min. The resulting lysates were centrifuged at 12,000 g, and a BCA Assay Kit (Beyotime) was employed for the quantification of protein samples. These samples were next boiled for 15 min, prior to separation *via* SDS-PAGE, then transferred onto PVDF membranes (Thermo Fisher Scientific). Subsequently, the blots underwent blocking in 5% non-fat milk in TBST, prior to an overnight exposure to primary antibodies (Supplementary Table S1) at 4°C, with subsequent rinses (3X) in TBST, incubation in secondary HRP-linked antibodies for 1 h, and assessment using chemiluminescent analysis (Bio-Rad). All samples were assessed using three replicates.

### Quantitative RT-PCR

TRIzol (Life Technologies, United States) was employed for total RNA extraction from DRG tissues, prior to cDNA synthesis from 1 µg RNA *via* the PrimeScript RT reagent kit (Takara, Dalian, China). Gene-specific transcript analyses were conducted with the MiniOpticon Real-Time PCR system (BioRad), and the transcript expressions were computed with the 2^−ΔΔCt^ formula. The qRT-PCR primers were made by Thermo Fisher Scientific, and are summarized in Supplementary Table S2.

### Histological analysis

The extracted sciatic nerves with SNI were sliced at twenty‐eight days post SNI. The sciatic nerves (0.5 cm anterior and posterior at injury site) underwent overnight fixation in cold 4% paraformaldehyde in 0.01 M phosphate‐buffered saline (PBS, pH = 7.4), prior to embedding in paraffin. Subsequently, longitudinal or transverse sections (5 μm thick) were cut for haematoxylin/eosin (HE) and immunofluorescence staining analyses, based on kit directions. Image capture was done under a light microscope.

### Walking track analysis

To explore rat behavior following SNI and repair, we conducted WTA at weeks 2 and 4 post operation. Rats were encouraged to step down a wooden alley (5.0 cm^3^ × 8.0 cm^3^ × 45 cm^3^) and reach a darkened goal box at the end. A white paper was laid on the alley floor. To track footprints, we applied a thin layer of acrylic paint to the rat’s plantar surface, which enabled us to visualize and record relevant anatomical footprint landmarks. Next, we utilized the walking paw prints to compute the sciatic functional index (SFI). The footprints were gathered to calculate SFI using the formula below:

SFI=(–38.3×(EPL−NPL)/NPL)+(109.5×(ETS−NTS)/NTS)+(13.3×(EIT−NIT)/NIT)−8.8

### Electrophysiological assays

At 4 weeks post-surgery, we performed electrophysiology examinations, as described in previous studies (Huelsenbeck et al., 2012). First, using isoflurane inhalation, we anesthetized rats, prior to exposing their corresponding sciatic nerves. A bipolar stimulating electrode was positioned on the proximal end of the nerve graft and shaved skin, 2 cm away from the stimulating cathode. Compound muscle action potentials (CMAPs) from the gastrocnemius belly on the ipsilateral side were measured *via* a multichannel electrophysiological recorder. The CMAP peak amplitude, nerve conduction velocity, and CMAP onset latency were next measured and compared across rat groups.

### Statistical analysis

In the present study, all data are provided as mean ± SEM, and analyses were done in GraphPad Prism (Version 8.0, San Diego, CA, United States) *via* one-way analysis of variance (ANOVA). Significance was tested using Tukey’s post hoc tests. Lastly, *p* < 0.05 was regarded as significant.

## Results

### Differentially expressed genes identification *via* the LIMMA package

Following the proper merging and removal of GSE58294 and GSE22255 batch effects, 987 DEGs were identified, among which, 445 were highly expressed, and 542 were scarcely expressed. A heatmap and volcano plot of the DEG results are presented in Supplementary Figures S1A,B.

### Identification of turquoise and BMs-Related genes

**FIGURE 1:**
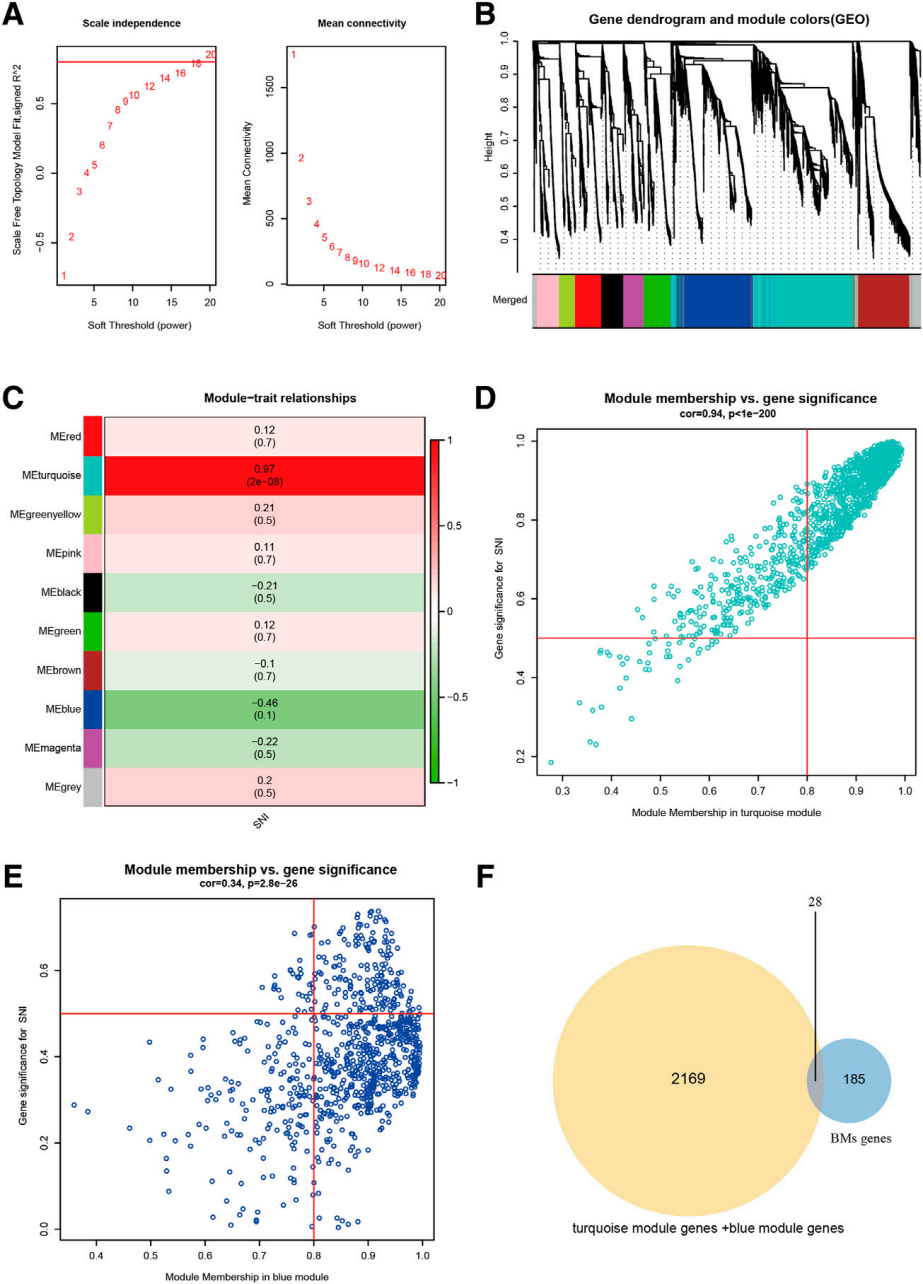
Screening of chief module and BM-linked genes. **(A)** Soft threshold setting with parameter R2 = 0.80. **(B)** The 6 modules-based separation of the gene set. **(C)** The modules versus SNI incidence association. **(D)** The turquoise module versus SNI gene significance correlation. **(E)** The blue module and SNI gene significance relationship. **(F)** The Venn plot of two gene sets, BMs, turquoise + blue module genes.

With the help of WGCNA, we screened for principal SNI-related modules. In Figure 1A, we assessed the scale-free fitting index (left) and mean connectivity (right) of multiple soft threshold powers, depending on scale-free *R* ^2^. In Figure 1B, all gene tree graphs were based on various metric (1-Tom) clustering. Individual tree branches represented a single gene, and each module color represented a co-expression module. The heatmap represented the association between the epigenome and SNI traits, with group-specific correlation coefficients and *p*-values (Figure 1C). Numbers in parentheses on the left represented the gene quantity in the subsequent epigenetic module. As illustrated in Figure 1C, the module with the largest positive association with SNI was the magenta module (r = 0.97), and the one with the largest negative association with SNI was the green module (r = −0.46). In addition, interaction analysis demonstrated that the association between the modules in turquoise and blue and SNI gene significance were 0.94 and 0.34, respectively (Figures 1D,E). The intersection of the two module gene sets and BMs-related genes is depicted in Figure 1F. Overall, 28 overlapping BM-related genes were screened for subsequent analysis.

### The sciatic nerve injury-Related biological functions included immune inflammatory responses and cell adhesion

**FIGURE 2:**
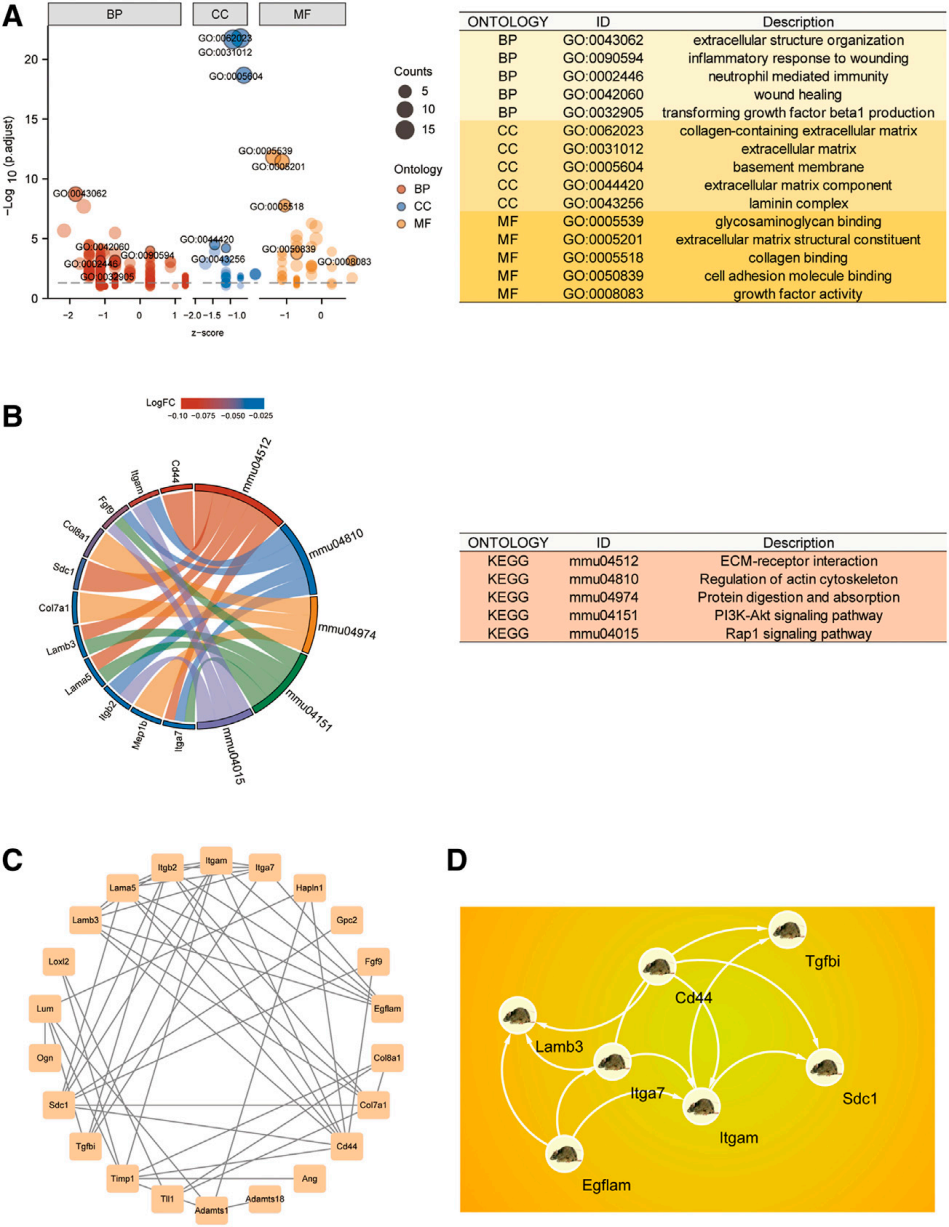
CG functional enrichment analyses (FEA) and hub gene identification. **(A)** GO FEA of CGs. **(B)** KEGG network analyses of CGs. **(C)** PPI network of CGs generated using the STRING database. **(D)** The leading 7 hub genes, as screened by MCC.

Using the “clusterProfiler” package, we generated a GO biological function enrichment analysis for these 28 BMs-related genes. In case of biological processes (BP), enrichments were found in the inflammatory response to wounding, extracellular structural organization, wound healing, transforming growth factor beta1 synthesis, and neutrophil-based immunity. In case of cellular component (CC), enrichments were in collagen-based ECM, BM, and laminin complex. In case of molecular functions (MF), enrichments were in collagen, cell adhesion molecule, and glycosaminoglycan interactions (Figure 2A). As depicted in Figure 2B, the KEGG network enrichment analysis revealed that 28 BM-related genes participated in ECM-receptor association, actin cytoskeletal modulation, and the PI3K-Akt axis. Taken together, the GO and KEGG analyses suggested that the SNI-related biological functions mostly involved immune inflammatory responses and cell adhesion.

### Hub gene identification using the protein–protein interaction network

To identify Hub differentially regulated genes, we conducted PPI network analysis on the 28 DEGs using STRING and Cytoscape (Figure 2C). The leading 7 positioning hub genes, namely, Itgam (Coding for the CD11b protein), SDC1, Egflam, Tgfbi, Lamb3, Cd44, and Itga7, were further examined (Figure 2D).

### The dorsal root ganglion immune status evaluation, based on CIBERSORT algorithms

**FIGURE 3:**
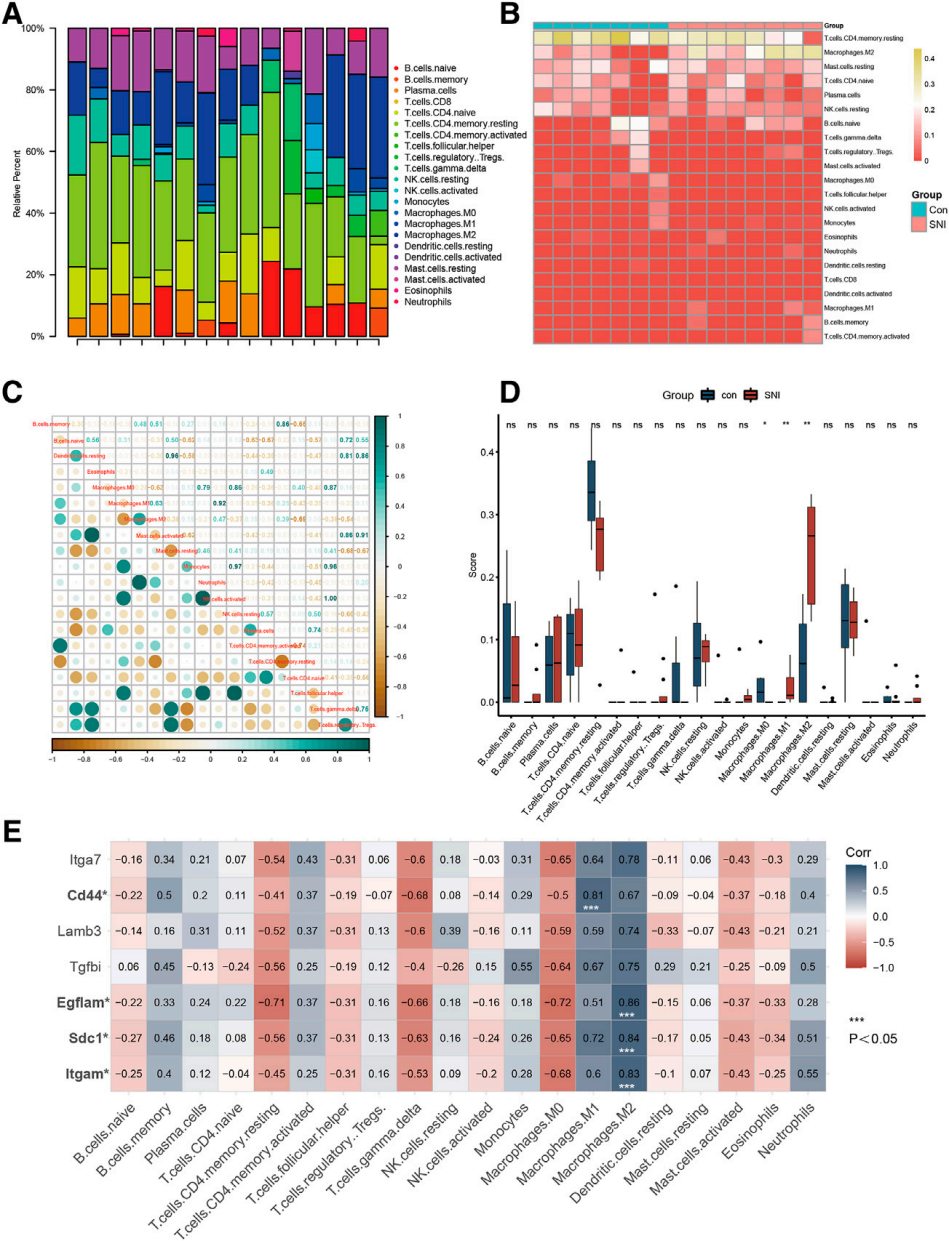
Immune infiltration analysis and immune-related CG Identification. **(A)** Heatmap of 28 immune genes in normal and SNI tissues, according to the CIBERSORT algorithm. **(B)** Heatmap of the 22 immune cell expressions in normal and SNI tissues. **(C)** Correlation heatmap of immune cells. **(D)** The profile difference of 20 immune cells between the SNI and healthy samples. **(E)** The profile difference of 7 hub genes in 20 immune cells.

Figures 3A,B depict a heatmap illustrating the 28 immune gene expressions in normal *versus* SNI tissues, as evidenced by the CIBERSORT algorithm. In terms of immune cells, M0 macrophages showed a significant inverse correlation (r = −0.28) with M1 macrophages. Alternately, M0 macrophages exhibited a marked negative association (r = −0.63) with M2 macrophage (Figure 3C). We next examined differences in the immune cell composition between SNI and sham DRG (Figure 3D). It was obvious that the M1 and M2 macrophage proportions were elevated in the SNI rats, compared to sham rats. Meanwhile, the M0 macrophage content in the sham rats were markedly increased, relative to the SNI rats. The 4 hub genes, Itgam, SDC1, Egflam, and CD44, were directly associated with immune cell invasion in DRG following SNI. CD44 was directly associated with M1 macrophage (*p* < 0.05), whereas, Itgam, SDC1, and Egflam were closely associated with M2 macrophages (Figure 3E) (*p* < 0.05).

### Sciatic nerve injury-induced neuro-inflammation in rats

**FIGURE 4:**
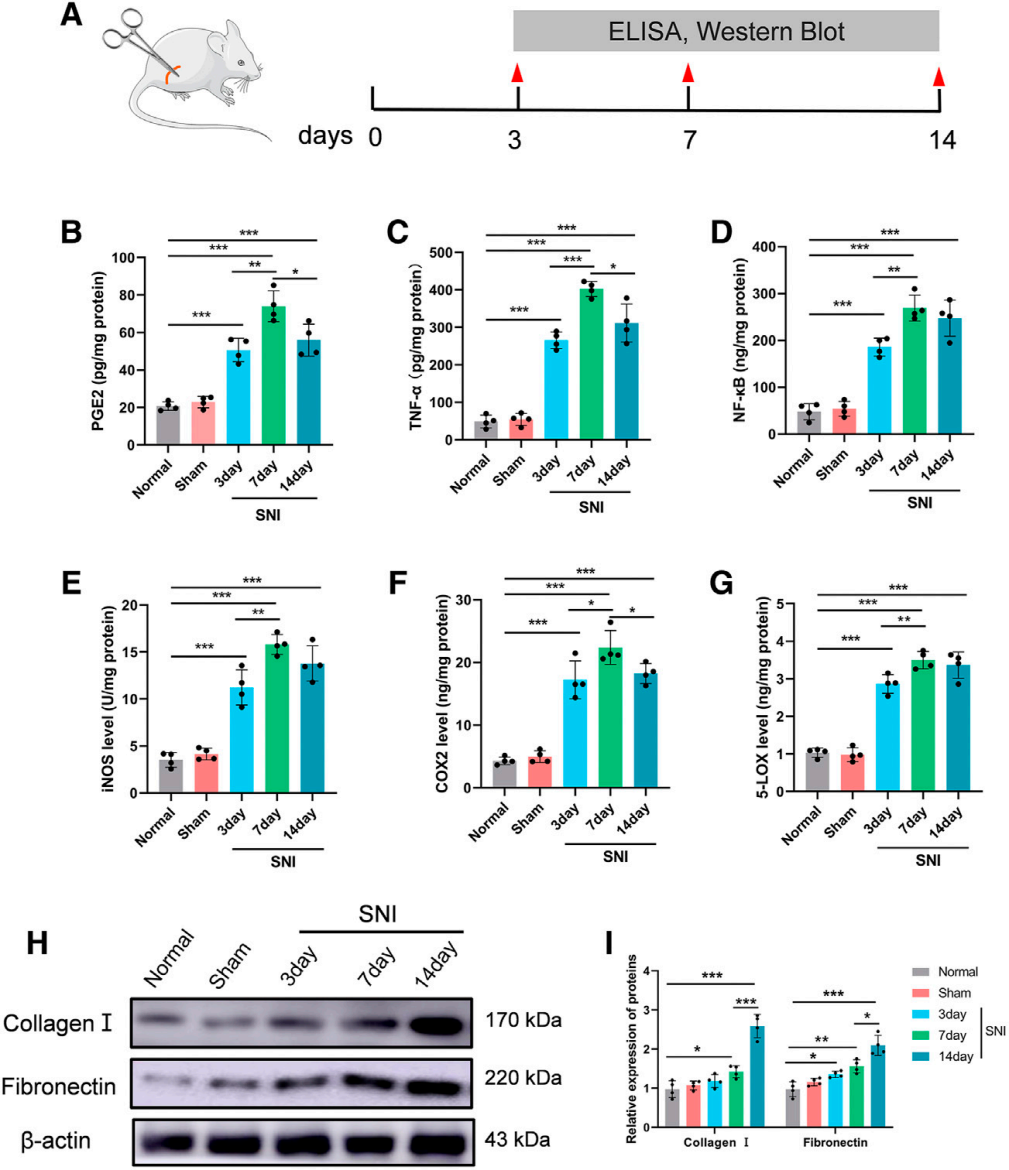
The level of inflammatory- and scar-related protein in the DRG. **(A)** Experimental paradigm. **(B)** PGE2, **(C)** TNF-α, **(D)** NF-κβ, **(E)** iNOS, **(F)** COX-2, and **(G)** 5-LOX in DRG tissues. **(H)** The expression of collagen I and fibronectin in DRG tissues at 0, 3, 7, and 14 days following injury, as evidenced by western blot analysis. **(I)** Protein data quantification from **(H)**. Data are provided as mean ± SD, with *n* = 4 per treatment. *p < 0.05, **p < 0.01, and ***p < 0.001 were deemed significant.

To assess the SNI-mediated inflammatory status in rats, the pro-inflammatory indicators PGE2, TNF-α, and NF-κB expressions, as well as COX-2 and 5-LOX enzymes, and the nitrosative stress enzyme iNOS were examined in DRG 3, 7, and 14 days after operation. Figure 4A depicts the experimental design of this study. Based on our analysis, all aforementioned parameters were increased in SNI rats, compared to sham rats. The inflammatory response was highest at 7 days post surgery, and began to diminish thereafter (Figures 4B–G). In addition, we also assessed CGs that associated with BM-related genes. Multiple investigations reported a strong role of inflammation in nerve adhesion etiology (Bu et al., 2020). Thus, we assessed collagen I and fibronectin contents following SNI in rats. Collagen I and fibronectin levels were dramatically augmented at 7 and 14 days in the SNI rats, and they reached their corresponding peaks at 14 days (Figures 4H,I).

### The hub gene targets Trichostatin A analysed by the chemical structures

**FIGURE 5:**
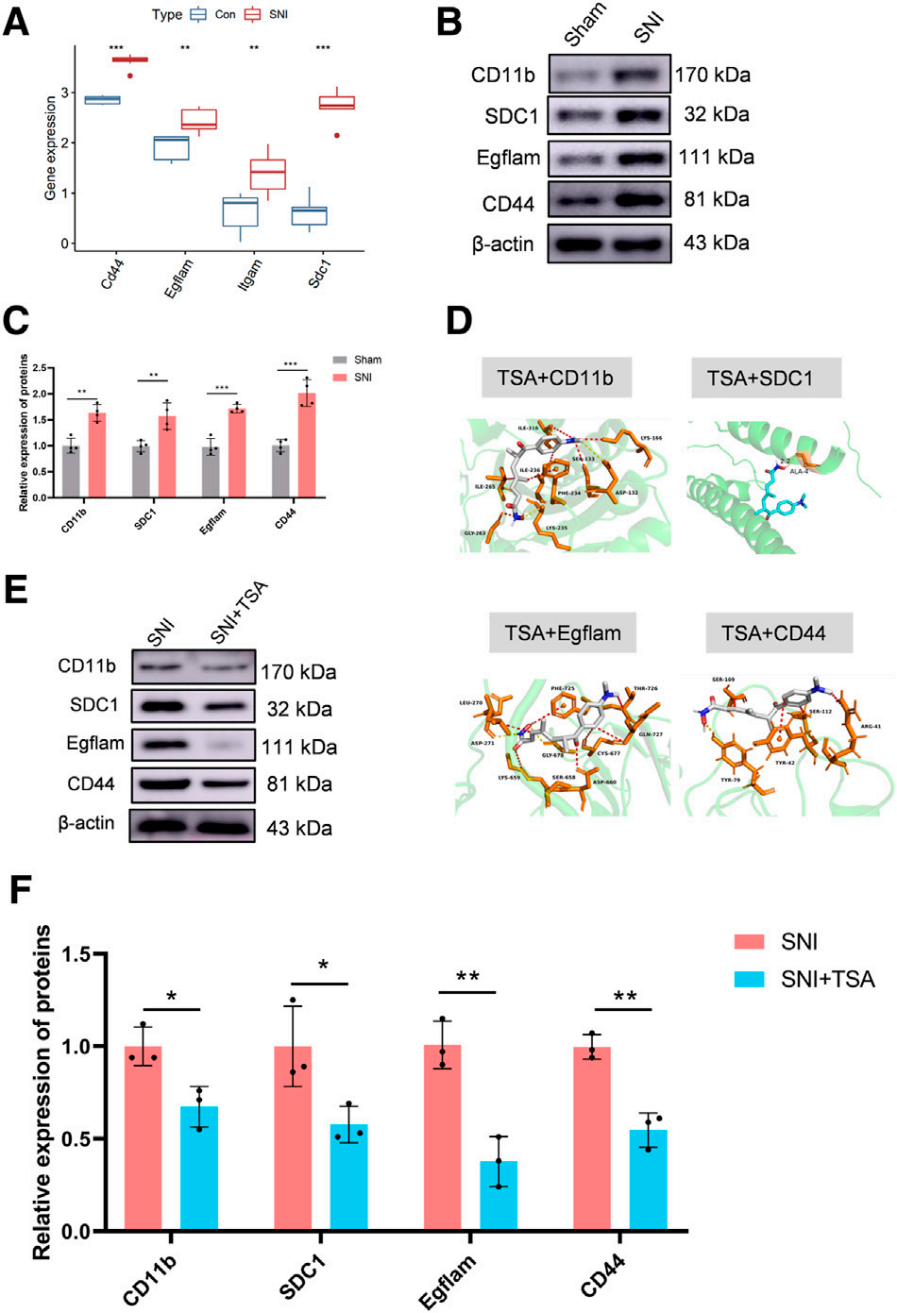
Validation of key genes and targeted drug prediction. **(A)** Expression of key genes in the GSE96051 and GSE161342 datasets. **(B)** Western blot was employed to verify key genes. **(C)** Protein data quantification from **(B)**. **(D)** Proteins and small molecular compound docking simulation. **(E)** Western blot was employed to verify the level of proteins in DRG after SNI. **(F)** Protein data quantification from **(E)**. Data are provided as mean ± SD, with *n* = 4 per treatment. **p < 0.01 and ***p < 0.001 are deemed significant.

In the GEO expression matrix, the Itgam, SDC1, Egflam, and Cd44 transcript levels in SNI rats were vastly upregulated, relative to sham rats (Figure 5A). We validated the key genes to enhance confidence of our results by constructing an SNI model in rats. Western blot was employed to determine hub gene expressions. Relative to sham rats, the Itgam, SDC1, Egflam, and Cd44 expressions were upregulated in SNI rats (Figures 5B,C).

The DSigDB database combines detailed drug information with extensive drug targets and activity data. The 4 identified genes were entered into the DSigDB database, and the targeted drugs were estimated. Our screening identified TSA as a potential drug that interacts with CD11b, SDC1, Egflam, and Cd44. Hence, we next examined the protein configurations of the CD11b, SDC1, Egflam and Cd44 proteins using the PDB database. Following binding energy calculations, TSA was shown to possess the highest binding energies with the aforementioned proteins (Figure 5D). To verify the prediction results, we test the protein level of CD11b, SDC1, Egflam, and Cd44 in DRG after SNI. Relative to SNI rats, the CD11b, SDC1, Egflam, and Cd44 expressions were downregulated in DRG after SNI (Figures 5E,F). However, more experiments are needed to confirm that TSA can interact with CD11b, SDC1, Egflam, and Cd44 *in vivo*.

### Trichostatin A inhibits neural inflammation, scarring, and enhances peripheral nerve regeneration following sciatic nerve injury

**FIGURE 6:**
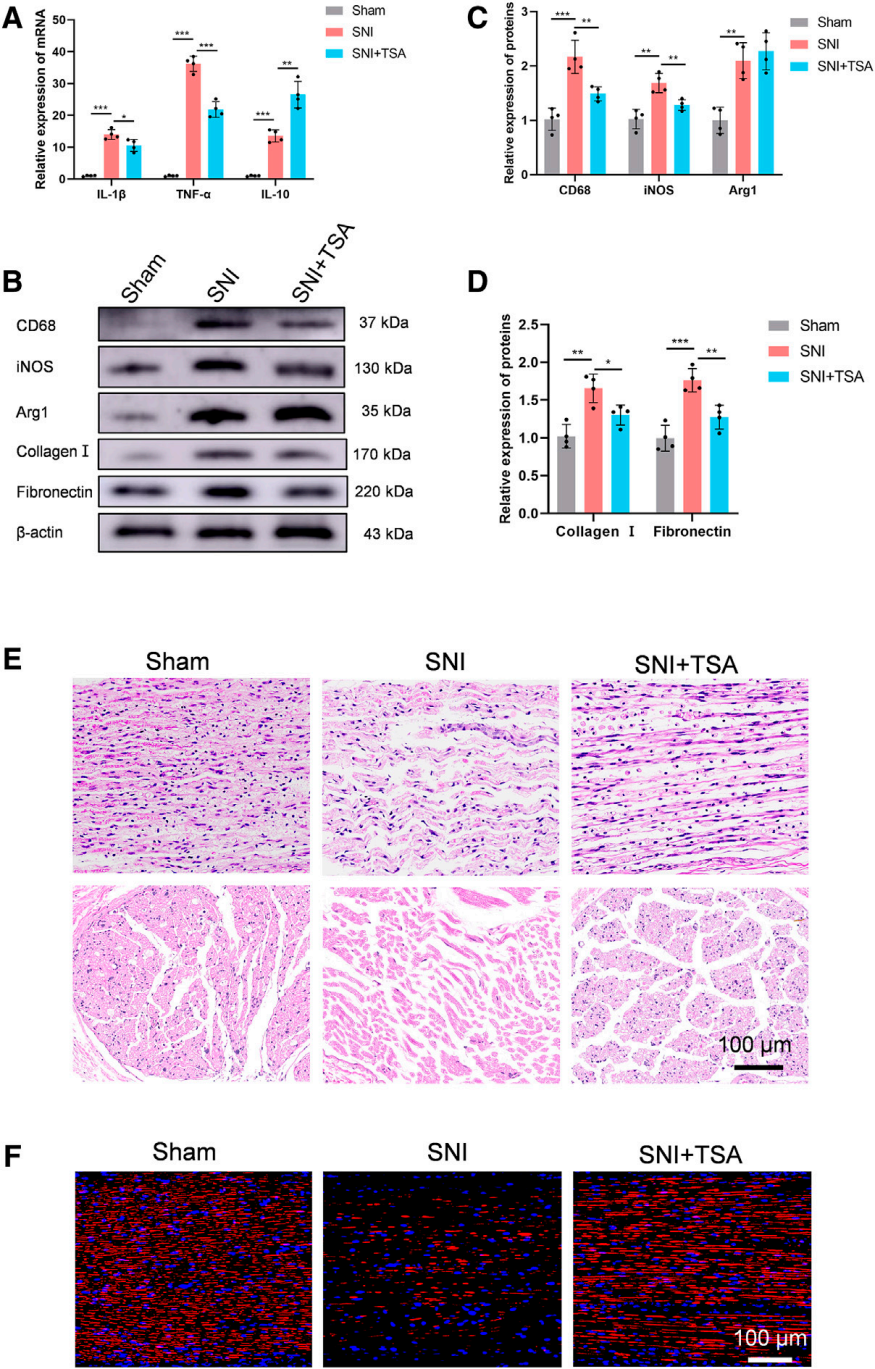
TSA inhibits neural inflammation and scarring and enhances PNR following SNI. **(A)** The relative IL-1β, TNFα, and IL-10 expression in DRG on day 14 post operation, as evidenced by real-time qPCR. **(B)** Protein expression in DRG tissues at day 14 post SNI, as evidenced by western blot analysis. **(C)** Protein data quantification from **(B)**. **(D)** Protein data quantification from **(B)**. **(E)** H&E staining of regenerated axons at the nerve segment in each group at 4 weeks post injury. **(F)** GAP43 (red) and DAPI (blue) staining of regenerated axons at the nerve segment of individual groups at 4 weeks post SNI. Data are provided as mean ± SD, with *n* = 4 per group. *p < 0.05, **p < 0.01, and ***p < 0.001 are deemed significant.

A recent study demonstrated that TSA enhances inflammatory response and liver injury in septic mice (Shen et al., 2022). To test the influence of TSA in mediating rat neural inflammation following SNI, we extracted rat DRG on the 14th day post operation. RT-PCR was used to quantify and characterize inflammatory cytokines in DRG. Based on our analysis, all cytokines were upregulated in adhesion *versus* normal tissues. In contrast, in SNI + TSA rats, the pro-inflammatory cytokines IL-1β and TNF-α contents were diminished, whereas the anti-inflammatory cytokine IL-10 was markedly enhanced, relative to SNI rats (Figure 6A). Moreover, CD68 (an activated monocytes/macrophage indicator), inducible-nitric oxide synthase (iNOS, a pro-inflammatory macrophage indicator), and arginase-1 (an anti-inflammatory macrophage indicator) were examined to screen for distinct macrophage categories *via* western blot. Based on our analysis, tissues from SNI rats showed a relatively large quantity of macrophage accumulation, particularly, the pro-inflammatory macrophage subtype. Alternately, tissues from SNI + TSA rats exhibited reduced macrophages, and most were the anti-inflammatory macrophage subtype, relative to SNI rats (Figures 6B,C).

We further validated tissue scarring by assessing collagen I and fibronectin levels *via* western blotting. Relative to sham rats, the collagen I and fibronectin expressions were substantially enhanced in SNI rats at 4 weeks, while TSA treatment dramatically diminished this SNI-mediated increase (Figures 6B,D).

We next explored the TSA-mediated effect on PNR, which is essential for functional recovery (FR) (Frendo et al., 2019; Liu et al., 2019). To further examine the histologic alterations within regenerated nerve fibers, H&E staining was conducted on longitudinal and transverse sections in all rat groups at 4 weeks post injury. Based on our analysis, the regenerated nerves fibers were relatively small and randomly arranged in the SNI rats, and TSA markedly improved this histological morphology (Figure 6E). We further assessed the TSA-mediated effect on PNR using NF‐200 (a heavy neurofilament subunit marking for both large and small axons) immunofluorescent staining on longitudinal sections. The amount of nerve fibers was markedly enhanced with TSA usage, as opposed to SNI rats (Figure 6F). Taken together, these findings suggested that TSA treatment aids in PNR following SNI.

### Trichostatin A induces motor and sensory functional recoveries following sciatic nerve injury

**FIGURE 7:**
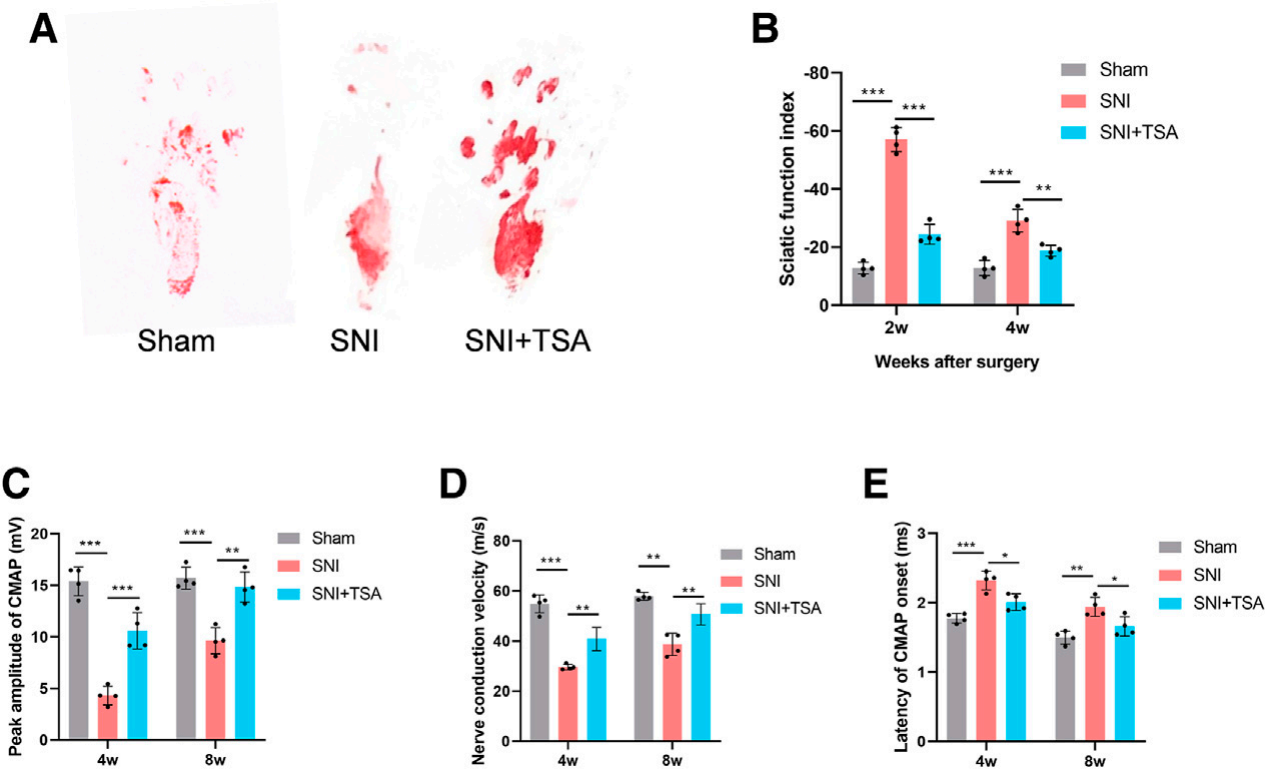
Functional evaluation of nerves. **(A)** Operative footprint images 4 weeks after surgery. **(B)** The SFI values of each group at 2 and 4 weeks after surgery. **(C–E)** Peak CMAP amplitude **(C)**, nerve conduction velocity **(D)**, and CMAP onset latency **(E)** 4 and 8 weeks postoperatively. Data are provided as mean ± SD, with *n* = 4 per group. *p < 0.05, **p < 0.01, and ***p < 0.001 are deemed significant.

To elucidate the TSA-mediated modulation of PNR, we assessed the locomoter recovery following TSA for 4 weeks, using WTA. Based on our analysis, SFI improved progressively with time. At 2 and 4 weeks post injury and treatment, the SFI in SNI + TSA rats was markedly enhanced, compared to SNI rats (Figures 7A,B), suggesting a better motor FR with TSA.

We next performed electrophysiological examinations to determine the 4- and 8-week FR of the repaired nerve following surgery. CMAP represents the evoked responses of the target muscle, and CMAP latency is described as the lag duration between the stimulation artifact and the first CMAP curve deflection onset. We revealed that the SNI + TSA rats exhibited an elevated CMAP peak amplitude, rapid nerve conduction velocity, and reduced CMAP latency, compared to the SNI rats (Figures 7I–K). These evidences suggested that TSA can achieve enhanced motor FR in rats following SNI.

## Discussion

With great advancements in next generation sequencing, bioinformatic analysis is quickly becoming the tool of choice for screening significant molecules in multiple diseases, including septic cardiomyopathy (Ivandic et al., 2012). Impaired BM protein expression and degradation are essential pathogenic factors that contribute to cancer, diabetes, and fibrosis (Tsilibary, 2003; Naba et al., 2014; Randles et al., 2021). Emerging evidences revealed a network of >200 BM-related factors that provide BM with an astonishing amount of complexities, which may be the direct cause of the wide diversity in BM functions that are essential for human health. The multidisciplinary explorative screening of BM-related factors, localization sites, principal BM modulatory nodes, and disease interaction highlights a strategy for the discovery of signaling pathways that dictate BM function and modulation. However, till date, the effect of BM components within peripheral nerves remains undetermined.

Herein, we integrated DEG profiling in SNI with BM analysis to generate PPI networks that enhance our comprehension of the SNI-based signaling pathways in DRG tissues, and develop potential therapeutic interventions. This is the first report that identified hub genes by integrating DEGs in SNI and BMs. In addition, we confirmed our results using a rat SNI model. This investigation offered a theoretical basis for the promising discovery of the underlying SNI-based signaling networks, treatment, and patient prognosis.

SNI is widespread in clinical settings, and, unfortunately, it is common for patients to experience nonreversible tissue atrophy and dysfunction, which, in turn, extolls a substantial burden on patients, their families, and societies (Johnson et al., 2005). Nerve inflammation strongly suppresses sciatic nerve regeneration (SNR) following SNI. In an animal model of SNI, Govindappa demonstrated strongly elevated pro-inflammatory genes (IL1β, iNOS, and CD68) and markedly diminished anti-inflammatory genes (IL10 and CD163), as well as the cell-surface marker CD206 at the site of injury (Govindappa and Elfar, 2022). However, prior investigations examined inflammation at the nerve injury site after SNI. A limited number of reports exists on the DRG tissue inflammation following SNI. A small number of innate and adaptive immune cells are present in the DRG, which increase in DRG accompany chronic pain conditions (Kim and Moalem-Taylor, 2011). In addition to neuroimmune interactions occurring at the site of injury, it has been shown that immune cells may also interact with the cell bodies of nociceptor neurons within the DRG to induce chronic pain. In addition, T cells also release leukocyte elastase in the DRG, which causes pain after nerve injury (Vicuña et al., 2015). In this report, to our surprise, the hub genes Itgam, SDC1, Egflam, and Cd44 were upregulated in both the M1 and M2 macrophages, and this inflammatory response was further confirmed using other experiments.

Inflammation mediated by nerve injury and surgery often results in PNAs with the surrounding tissue (Akbar et al., 2020). Herein, we demonstrated that the neuroadhesin levels were markedly elevated in DRG tissues following SNI. Even though multiple cell types contribute to pathological fibrosis, macrophages typically possess essential modulatory activities in all stages of repair and maintenance (Mosser and Edwards, 2008). Numerous studies independently approached the prevention or inhibition of PNA. However, macrophage-targeting is not fully elucidated (Yamamoto et al., 2010). Hence, novel therapeutic approaches targeting inflammation, particularly macrophage-related networks, may be beneficial to the prevention of nerve adhesion.

Histone deacetylases (HDACs) belong to an enzyme family that modulates gene expression *via* alteration of the acetylation status of DNA-associated histone and nonhistone proteins (Beckerman et al., 2014). Emerging reports examined the relevance of post-translational histone modifications in the determination of macrophage polarization progression (Ivashkiv, 2013). It was revealed that TSA (an HDAC inhibitor) administration augmented M2 macrophage concentration, while attenuating renal fibrosis (Tseng et al., 2020). Nevertheless, the significance of epigenetic modulation on macrophage phenotypes in DRG remains undetermined following SNI. Herein, we reported that TSA strongly inhibited M1 macrophage invasion, augmented M2 macrophage concentration, and reduced fibrosis in DRG following SNI. We speculated that TSA modulated an M1-to-M2c switch. The TSA-mediated increase in M2 macrophages was strongly associated with diminished DRG fibrosis. Moreover, TSA also abrogated fibrotic and inflammatory phenotypes. Taken together, these findings suggested that TSA diminished DRG fibrosis *via* an increase in M2 macrophage expression.

Interestingly, in addition to promoting nerve regeneration by inhibiting scarring, TSA also enhanced levels of IL-10 in DRG. IL-10 is reported to be highly beneficial for nerve regeneration (Atkins et al., 2007). In this report, we revealed that TSA administration promoted elevated IL-10 expression, which is a strong indicator of Mregs (Mosser and Edwards, 2008). Unlike wound-healing macrophages, Mregs are robust inflammation suppressors which produce arginase-1 to suppress fibrosis, however, they do not participate in ECM synthesis (Murray and Wynn, 2011; Chandrasekaran et al., 2019). Relative to SNI rats, arginase-1 levels were enhanced in the SNI + TSA rats. Over the past decade, the TSA-mediated anti-adhesion effect is still under question (Tseng et al., 2020). Herein, we demonstrated a strong anti-inflammatory and anti-adhesion property of TSA, which was achieved by polarizing macrophages to certain regulatory types. Since this investigation was based on systemic drug administration, further evaluation of pharmacokinetics is highly warranted.

**FIGURE 8:**
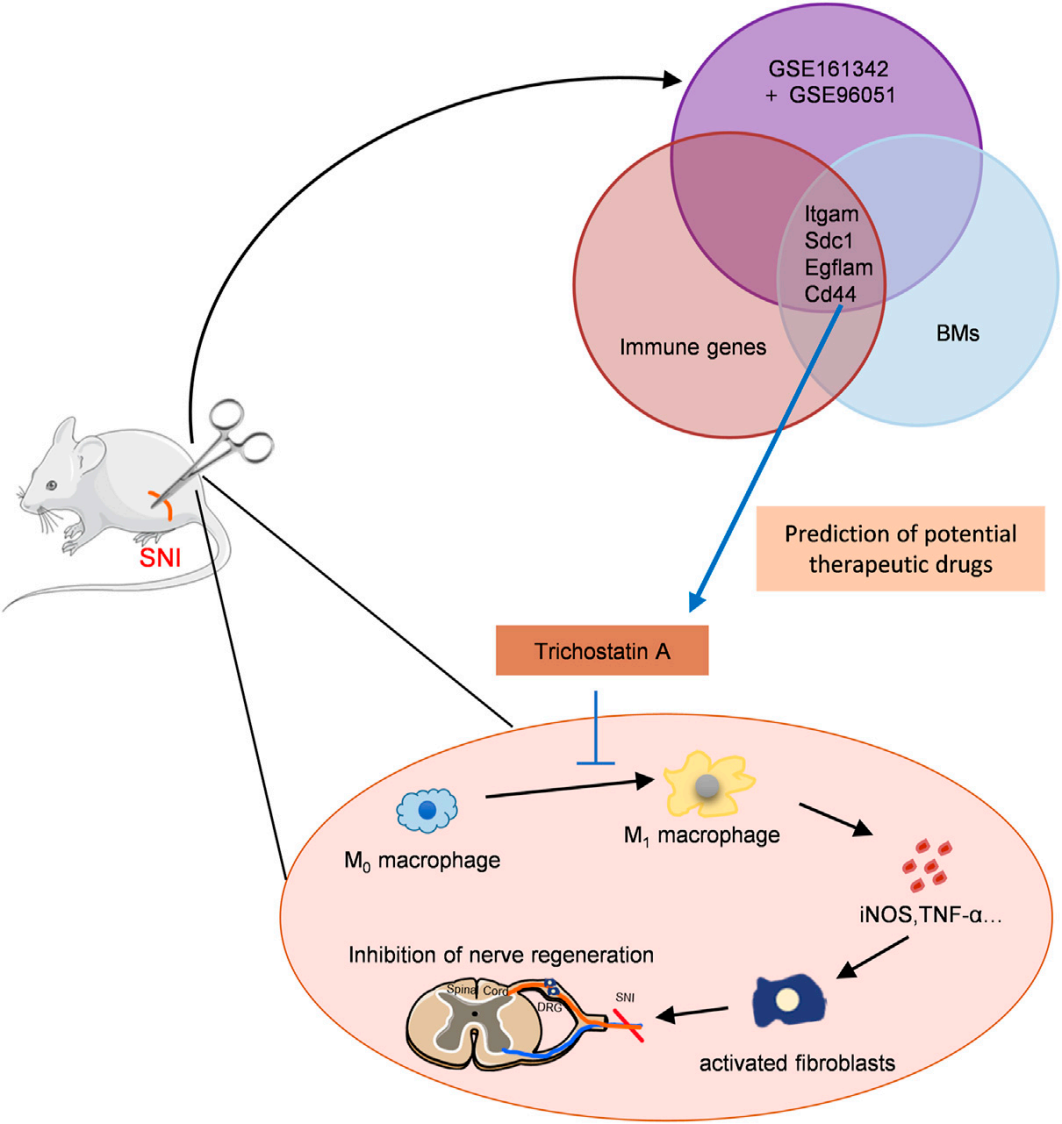
Schematic model showing discovery and validation of novel immunotherapeutic drug candidates for sciatic nerve injury (SNI). The key immune-related CGs were identified *via* PPI and immune infiltration analyses. TSA was predicted as the targeted drugs of these genes by the DSigDB database. TSA treatment promoted M2 macrophage polarization, and augmented IL-10 release while decreasing IL-1β and TNFα levels, thus, enhancing axonal regeneration.

As illustrated in Figure 8, this is the first report that suggested that TSA improves PNA following SNI using bioinformatic analysis and experimental verification. TSA exposure induced M2 macrophage polarization, which further augmented IL-10 release and diminished IL-1β and TNFα expressions. We also demonstrated that TSA not only enhanced axonal regeneration but also promoted FR following SNI. These findings provide novel ideas into the TSA-mediated enhancement of PNR, and highlights its therapeutic potential in nerve injury repair.

## Data Availability

The original contributions presented in the study are included in the article/Supplementary Materials, further inquiries can be directed to the corresponding authors.
