# Supplementary material for: Discovery of novel immunotherapeutic drug candidates for sciatic nerve injury using bioinformatic analysis and experimental verification
Source: Front Pharmacol. 2022 Nov 7;13:1035143. doi: 10.3389/fphar.2022.1035143 (PMC9676506; doi:10.3389/fphar.2022.1035143)
Supplement: Supplementary file 1 [file Table1.DOCX]

Supplementary Table 1: Antibody information

| Name | Cat. | Company | Country |
| --- | --- | --- | --- |
| Anti-CD11b | ab133357 | Abcam | UK |
| Anti-SDC1 | ab128936 | Abcam | UK |
| Anti-EGFLAM | ab101398 | Abcam | UK |
| Anti-CD44 | ab243894 | Abcam | UK |
| Anti-CD68 | ab283654 | Abcam | UK |
| Anti-iNOS | GB11119 | Servicebio | China |
| Anti-Arginase 1 | GB11285 | Servicebio | China |
| Anti-Collagen I | GB13022-2 | Servicebio | China |
| Anti-Fibronectin | ab268020 | Abcam | UK |
| Anti-GAP43 | ab75810 | Abcam | UK |
| Anti-β-actin | GB15001 | Servicebio | China |

Table S2: primer sets used for RT-PCR

| Primer | Sequence (5'-3') |
| --- | --- |
| IL-1β Forward primer | AATCTCACAGCAGCATCTCGACAAG |
| IL-1β Reverse primer | TCCACGGGCAAGACATAGGTAGC |
| TNFα Forward primer | AAAGGACACCATGAGCACGGAAAG |
| TNFα Reverse primer | CGCCACGAGCAGGAATGAGAAG |
| IL-10 Forward primer | GGCAGTGGAGCAGGTGAAGAATG |
| IL-10 Reverse primer | TGTCACGTAGGCTTCTATGCAGTTG |
| β-actin-Forward primer | TAGTTGCGTTACACCCTTTCTTG |
| β-actin-Reverse primer | TCACCTTCACCGTTCCAGTTT |


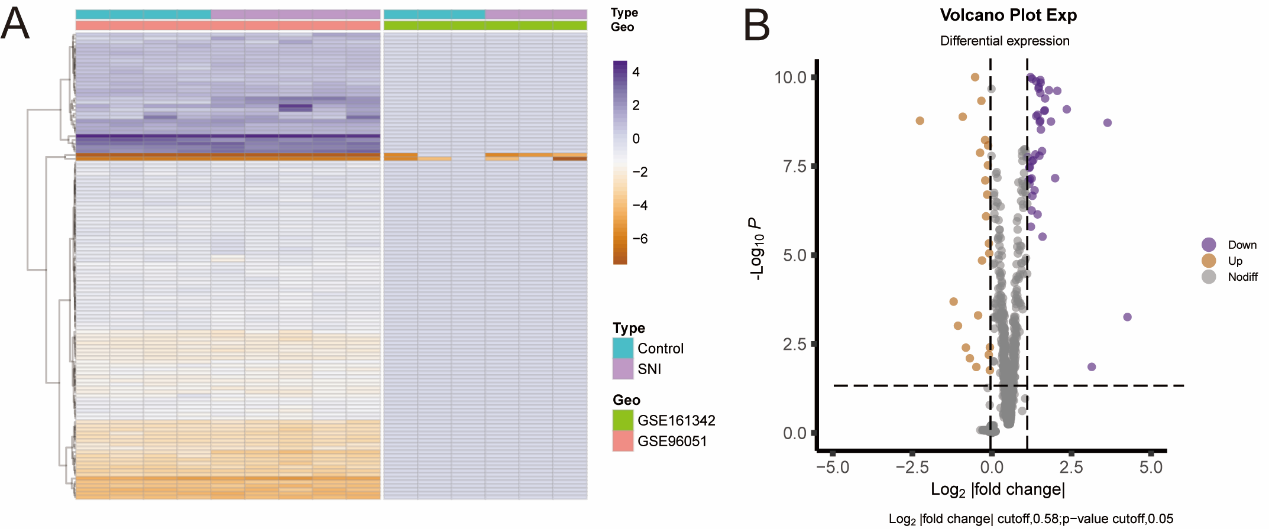


Figure S1: (A) Heatmap of differentially expressed genes. Purple means high expression, brown means low expression. (B) Volcano map of differentially expressed genes.
